# Latex proteins downregulate inflammation and restores blood-coagulation homeostasis in acute *Salmonella* infection

**DOI:** 10.1590/0074-02760200458

**Published:** 2020-11-23

**Authors:** Brandon Ferraz Sousa, Ayrles Fernanda Brandão da Silva, José Vitor Lima-Filho, Anderson Gomes Agostinho, Denise Nunes Oliveira, Nylane Maria Nunes de Alencar, Cleverson Diniz Teixeira de Freitas, Márcio Viana Ramos

**Affiliations:** 1 Universidade Federal do Ceará, Departamento de Bioquímica e Biologia Molecular, Fortaleza, CE, Brasil; 2 Universidade Federal Rural de Pernambuco, Departamento de Biologia, Recife, PE, Brasil; 3 Universidade de Fortaleza, Departamento de Patologia, Fortaleza, CE, Brasil; 4 Universidade Federal do Ceará, Departamento de Fisiologia e Farmacologia, Fortaleza, CE, Brasil

**Keywords:** cytokines, coagulation, haemostasis, nitric oxide, peritoneal cavity

## Abstract

**BACKGROUND:**

*Calotropis procera* latex protein fraction (LP) was previously shown to protect animals from septic shock. Further investigations showed that LP modulate nitric oxide and cytokines levels.

**OBJECTIVES:**

To evaluate whether the protective effects of LP, against lethal bacterial infection, is observed in its subfractions (LP_PII_ and LP_PIII_).

**METHODS:**

Subfractions (5 and 10 mg/kg) were tested by i.p. administration, 24 h before challenging with lethal injection (i.p.) of *Salmonella* Typhimurium. LP_PIII_ (5 mg/kg) which showed higher survival rate was assayed to evaluate bacterial clearance, histopathology, leukocyte recruitment, plasma coagulation time, cytokines and NO levels.

**FINDINGS:**

LP_PIII_ protected 70% of animals of death. The animals given LP_PIII_ exhibited reduced bacterial load in blood and peritoneal fluid after 24 h compared to the control. LP_PIII_ promoted macrophage infiltration in spleen and liver. LP_PIII_ restored the coagulation time of infected animals, increased IL-10 and reduced NO in blood.

**MAIN CONCLUSIONS:**

LP_PIII_ recruited macrophages to the target organs of bacterial infection. This addressed inflammatory stimulus seems to reduce bacterial colonisation in spleen and liver, down regulate bacterial spread and contribute to avoid septic shock.

The latex exuded by laticifer plants plays an important role in plant defense against pathogens and herbivores. Secondary metabolites, proteins and peptides, present in laticifer fluids, are expected to act against an incoming infection or predation. This action-specificity means that each group of chemical compounds that composes latex fluids possesses a specific molecular target, inside or outside the plant.^1^

For a long time, different parts of the plant *Calotropis procera* (Aiton) WT Aiton, are being studied for their use in alternative medicine, aiding the treatment of various conditions, including pain, asthma, eczema, mouth bleeding and superficial skin burns.^2^ More importantly, the latex from *C. procera* has been studied due to its effects over cellular immunity on various models. These effects include analgesic, anti-inflammatory, proinflammatory, antimicrobial activity, and others.^3^ ^,^ ^4^

Among the constituents of *C. procera* latex, approximately 85% of its dry mass is rubber (polyisoprene), while soluble proteins represent around 10%. Among the soluble laticifer proteins (LP), are peptidases, that degrade substate BANA, and azocasein in neutral pH.^5^ The fractionation of the *C. procera* LP, through CM-Sepharose *Fast Flow* chromatography and further biochemical characterisation of each obtained subfraction, lead to a better understanding of the composition of the plant’s latex proteins, and allowed a more in-depth study of its capacity to affect cellular immunity.

LP_PI_, LP_PII_ and LP_PIII_ are the subfractions resulting of LP chromatography.^6^ LP was shown to modulate the immune response in various animal models, by reducing histamine leakage, nitric oxide (NO) synthesis, and cytokines release, allowing a complex balance among anti-inflammatory and proinflammatory activities depending of specific physiological circumstances.^7^ ^,^ ^8^

*Calotropis procera* LP was studied for their effect on bacterial infection of human interest. LP did not inhibit growth of *Salmonella enterica enterica* serovar Typhimurium *in vitro*, thus, this fraction does not act directly on the bacterium. However, when mice were treated by different inoculation routes, the dose of 60 mg/kg of LP given intraperitoneally, fully protected animals against the lethal *Salmonella*, whereas the administration by oral and subcutaneous routes did not protect the animals. Although the treatment with LP prevented the death of the animals, the study reported that bacteria were still present in liver and spleen, causing transient histological damage. The presence of bacteria in bloodstream and target organs of treated animals suggested that LP modulate inflammation, avoiding septic shock.^9^ Further, bacteria clearance was reached after 28 days.

When assessing the influence of LP over the coagulation haemostasis, the intraperitoneal administration of LP abrogated the coagulant reaction caused by *Salmonella* infection. However, there is no information whether the protective effect of LP against septic shock is restricted to LP_PI_, LP_PII_ and LP_PIII_ subfractions or whether synergisms may occur.

In another study, the three subfractions LP_PI_, LP_PII_ and LP_PIII_ were tested for their effect on blood coagulation. The subfractions LP_PII_ and LP_PIII_ that exhibit proteolytic activity, demonstrated procoagulant activity over human plasma. These two subfractions also exhibited fibrinogenolytic and fibrinolytic effects. Both effects were not observed in LP_PI_ that does not possesses peptidases. Worth of note, LP restored to near normality the coagulation time of blood in infected animals. This effect inhibited the disseminated intravascular coagulation, a phenomenon observed in lethal bacteria infection. This study suggested that the peptidases present in *C. procera* latex possess biological activities that may be useful for treatment of coagulation abnormalities.^10^

The first attempt to investigate the role of LP subfractions in protection against bacterial infection was performed with LP_PI_. The subfraction LP_PI_ was tested against the same *Salmonella* infection model and some aspects of its action over the immune system was investigated. Animals treated by intraperitoneal route survived the infection challenge. Similar to the LP treatment, LP_PI_ induced high bacterial clearance in the bloodstream. mRNA of TNF-α was increased early in treated animals, alongside a reduction in NO contents in the blood. Neutrophil infiltration into the peritoneal cavity was enhanced and it was concluded that this inflammatory stimulus, caused by the LP_PI_ administration, may induce phagocytosis of bacteria, affording protection against animal death.^6^ However, the mechanism underlying this effect remains unknown.

Although the mechanisms of LP action remain obscure, the ability of this preparation to modulate inflammation has been proved in other relevant inflammatory conditions, including arthritis and chemotherapy.^11^ Given this information, LP is shown to be a protein fraction of great pharmacological interest due to its activity over the immune system and inflammation. To better understand the protective effect of LP over the bacterial infection, in this study, the subfractions LP_PII_ and LP_PIII_ were examined. Some aspects of the effectiveness of subfractions LP_PII_ and LP_PIII_ were investigated against the same infection model, focusing on the one that yielded the best survival rates in challenge experiments.

## MATERIALS AND METHODS

*Latex extraction and processing* - Vegetative and reproductive samples of *C. procera* shrubs were identified by specialists of the Herbarium Prisco Bezerra located at the Federal University of Ceará and the materials were deposited under voucher number 32663. The access and use of this biological resource were performed after registration and legal authorisation according to the current Brazilian law for biodiversity (Agreement nº A689147). The latex was collected fresh, following previous methodologies.^7^ *C. procera* LP were obtained after centrifugation, dialysis and lyophilisation, of the latex. Aiming to inhibit the proteolytic activity of the LP, iodoacetamide (IAA) was used accordingly.^12^ Thus, the latex was also collected on 10 mM IAA solution, instead of distilled water.

*Chromatography fractionation* - *C. procera* LP was fractionated through ion-exchange chromatography. A CM-Sepharose Fast Flow matrix was used and equilibrated with 50 mM sodium acetate buffer (pH 5.0). LP (10 mg/mL) was dissolved in the same buffer, followed by centrifugation to remove insoluble material. After loading the sample, the chromatography system was washed with the initial buffer and LP_PI_ was recovered. The adsorbed fractions were eluted by increasing the ionic strength of the buffer by adding 0.2 M and 0.3 M of sodium chloride (NaCl), sequentially. Fractions of 2 mL were collected at 0.5 mL/min flow rate. Each fraction had its absorbance read at 280 nm in a spectrophotometer. Two distinct protein peaks were obtained within salt gradient (LP_PII_ and LP_PIII_), respectively. The processing and fractionation of the latex collected with IAA were performed exactly as described above, in this case the samples were labeled LP_PII_-IAA and LP_PIII_-IAA.^10^ All the samples were dialysed in distilled water followed by freeze-dry before use. The fractions treated with IAA were checked for absence of proteolysis according to Freitas et al.^5^

*Protein profile analysis by sodium dodecyl sulphate-polyacrylamide gel electrophoresis (SDS-PAGE)* - One dimensional electrophoresis was used to accompany the fractionation steps. Electrophoresis were proceeded according to standard procedures.^13^ Molecular mass markers were used for reference phosphorylase β (97.0 kDa), bovine serum albumin (66.0 kDa), ovalbumin (45.0 kDa), carbonic anhydrase (30.0 kDa), trypsin inhibitor (20.1 kDa) and α-lactalbumin (14.4 kDa). The runs were executed in constant current of 30 mA per gel and tension of 110 V per gel, in an average duration of 2 h at 25ºC. In each lane, 30 µL of each sample diluted to 1 mg/mL in distilled water was applied. Protein bands were visualised after coloring with Coomassie Brilliant Blue R-250.

*Ethics and animals* - Adult male Swiss mice (*Mus musculus*), weighing between 30 and 35 grams, obtained from the Central Animal House of the Federal University of Ceará were used. They were kept in cages, in a room with controlled air temperature of 24ºC and 12-h day-night cycle, with free access to water and food (standard commercial food from Purina, Paulínia, SP, Brazil). The animals were handled by strictly following the recommendations from the National Council for the Control of Animal Experimentation - CONCEA, after approval by the local institution’s Ethic Committee on the Use of Animals (CEUA - UFC, under protocols 5786300718 and 9112020519).

*Microorganisms* - The systemic infection of Swiss mice was provoked by *Salmonella enterica enterica* serovar Typhimurium C5 strain, which was a gift by Dr Pietro Mastroeni from Cambridge University, UK. The bacteria were activated in Brain Heart Infusion broth at 37ºC for 18 h and then cultured in brain heart infusion (BHI) agar for another 24 h at 37ºC. For bacterial enumeration, after animal infection, samples from infected animals were plated and bacteria were counted at MacConkey agar incubated at 37ºC for 24 h.

*Survival evaluation* - To evaluate the protective effect of *C. procera* subfractions, against the bacterial infection model, animals, in experimental groups, received a single administration of 0.2 of LP_PII_, LP_PII_-IAA, LP_PIII_ or LP_PIII_-IAA (5.0 and 10.0 mg/kg), diluted in phosphate-buffered saline (PBS, pH 7.2), by intraperitoneal route (i.p). The infection model with *Salmonella* Typhimurium was executed as described by Lima-Filho et al.^9^ Thus, 24 h after the protein administration, the animals were challenged by inoculation of a bacterial suspension of *S.* Typhimurium on 0.2 mL of sterile PBS via i.p. The bacterial suspension was prepared by diluting previously activated and isolated colonies of *S.* Typhimurium in PBS until attaining a bacterial suspension of 10^8^ colony-forming units per milliliter (equivalent of 0.5 absorbance units of optical density at 600 nm), which was diluted 100-fold achieving 10^6^ colony-forming unit (CFU)/mL before inoculation. For survival evaluation, a group of animals received only PBS before infection to serve as control, all animals (n = 10 per group) were infected at once using the same bacterial suspension. After inoculation, all groups were observed daily up to 7 days. Behavioral alterations, such as changes in posture, mobility, facial expression, grooming and vocalisation and distress were evaluated. The treatment that showed the best survival rate continued to be used in the following experiments. The results were expressed as percent survival of each group along seven days, a log-rank test was used to assess statistical differences.

*Experimental design* - The following analyses were done using LP_PIII_ at the dose of 5 mg/kg. Thus, the animals were randomly divided into four groups. Two PBS groups were formed. One PBS group (n = 6) consisted of animals that only received 0.2 mL of PBS i.p. The other PBS group (n = 12) was formed with animals that received PBS i.p. and were infected 24 h later. Similarly, two LP_PIII_ groups were formed, a first one (n = 12) that consisted of healthy animals that only received LP_PIII_ i.p. The other group (n = 12), animals received LP_PIII_ i.p. and were infected 24 h later. Except for the healthy PBS group, all other groups had half of their animals euthanised 24 h after inoculation/administration (n = 6) and the other half were euthanised 72 h post inoculation/administration (n = 6). The groups that were infected, received an intraperitoneal inoculation of bacterial suspension (0.2 mL, 10^6^ CFU/mL in PBS). This design was repeated three times under the same conditions, every time different materials were collected to run different analysis, always respecting the ethics by executing as most assays as possible with the least number of animals.

*Bacterial enumeration* - Infected mice were anesthetised, by administering 0.2 mL of a solution of Xylazine (10 mg/kg) and Ketamine (100 mg/kg) by intraperitoneal route. Blood samples of approximately 800 µL were collected from the retro-orbital plexus of each animal under anesthesia, followed by euthanasia. Afterwards, the abdominal cavity was opened, and the liver and spleen were aseptically removed. Peritoneal fluid was obtained by washing the abdominal cavity with 3 mL of sterile PBS containing 1% ethylenediaminetetraacetic acid (EDTA). All analyses were done individually from the material collected from each animal. Bacterial enumeration in the blood, fluid and organs was done after dilution in PBS and aliquots were plated onto MacConkey agar for 24 h of incubation at 37ºC. Results were expressed as CFU per gram of organ or milliliter of blood. One-way analysis of variance (ANOVA) was employed with Bonferroni post-hoc test to evaluate statistical differences.

*Cells count* - Blood and peritoneal fluid collected as described above was used to count leukocytes as previously reported by Souza and Ferreira.^14^ Total number of leukocytes was counted followed by differential count of neutrophil cells. The results were expressed as 10^3^ cells per milliliter of plasma or fluid. ANOVA was employed with Bonferroni post-hoc test to evaluate statistical differences.

*Histological analyses* - After liver and spleen removal, a small section of the organs was immersed into 10% buffered formaldehyde (pH 7.2) for fixation. This material was dehydrated and diaphanised before inclusion in paraffin for cutting slices of 5 µm thickness in a microtome, to be mounted into microscope glass slides. The microscope slides were dyed with hematoxylin-eosin, for analysis of inflammatory infiltration and general damage. A histological report of each group was elaborated after a blind unbiased reading of at least three different slices of every organ of each animal, by a pathologist, in look for signs of organ architecture damage and presence of inflammatory infiltrate.

*Measuring cytokines in plasma and peritoneal fluid* - The measurement of TNF-α, IL-1β and IL-10 in blood plasma and peritoneal fluid was performed only in animals euthanised within 24 h, due to experimental constrains. Mouse enzyme-linked immunosorbent assay (ELISA) kits were used (R&D Systems), the assays were performed strictly following manufacturer’s instructions. ANOVA was employed with Bonferroni post-hoc test to evaluate statistical differences.

*Measuring of NO in plasma and peritoneal fluid* - The levels of NO in plasma and peritoneal fluid samples were calculated indirectly, by measuring nitrate concentrations, through the conversion of nitrate to nitrite, by means of the enzyme nitrate reductase.^15^ The plasma and peritoneal fluid were obtained as previously described. The material was kept on ice for up to 8 h, when the assay was executed. The samples were incubated with KH_2_PO_4_ buffer (pH 7.5) containing nitrate reductase, in 96-well plates. A standard curve of sodium nitrate was processed alongside the samples. After 12 h, the Griess reagent was added to the plate. The absorbances were read at 560 nm. The results were expressed as µM of NO^3-^/NO^2-^. ANOVA was employed with Bonferroni post-hoc test to evaluate statistical differences.

*Plasma coagulation time* - The blood of the previously described groups was collected in 0.11 M sodium citrate and centrifuged at 500 g for 15 min (20ºC) to obtain the plasma. The clot formation was induced by addition of 30 µL of 0.25 M CaCl_2_ to the plasma. The coagulant activity was assessed by measuring the time between the addition of calcium chloride and the formation of a visible clot in the samples. Prothrombin time (PT) and activated partial thromboplastin time (aPTT) haemostasis tests were used (LABTEST Ref.: 501 and 502 respectively), and manufactures instructions were followed accordingly. Coagulation time was measured in a coagulometer (QUICK-TIME, DRAKE). Results were expressed as coagulation time in seconds.

*Statistical analyses* - All statistical analyses were done using GraphPad Prism software version 8.0.2. Survival experiments were analysed using Mantel-Cox log rank test. For all the other experiments, ANOVA was employed with multiple comparison post hoc Bonferroni corrections. Significant differences were noted when p < 0.05 in all tests. In all cases, results are the mean ± standard error of mean of at least three independent experiments or measurements.

## RESULTS

**Fig. 1:**
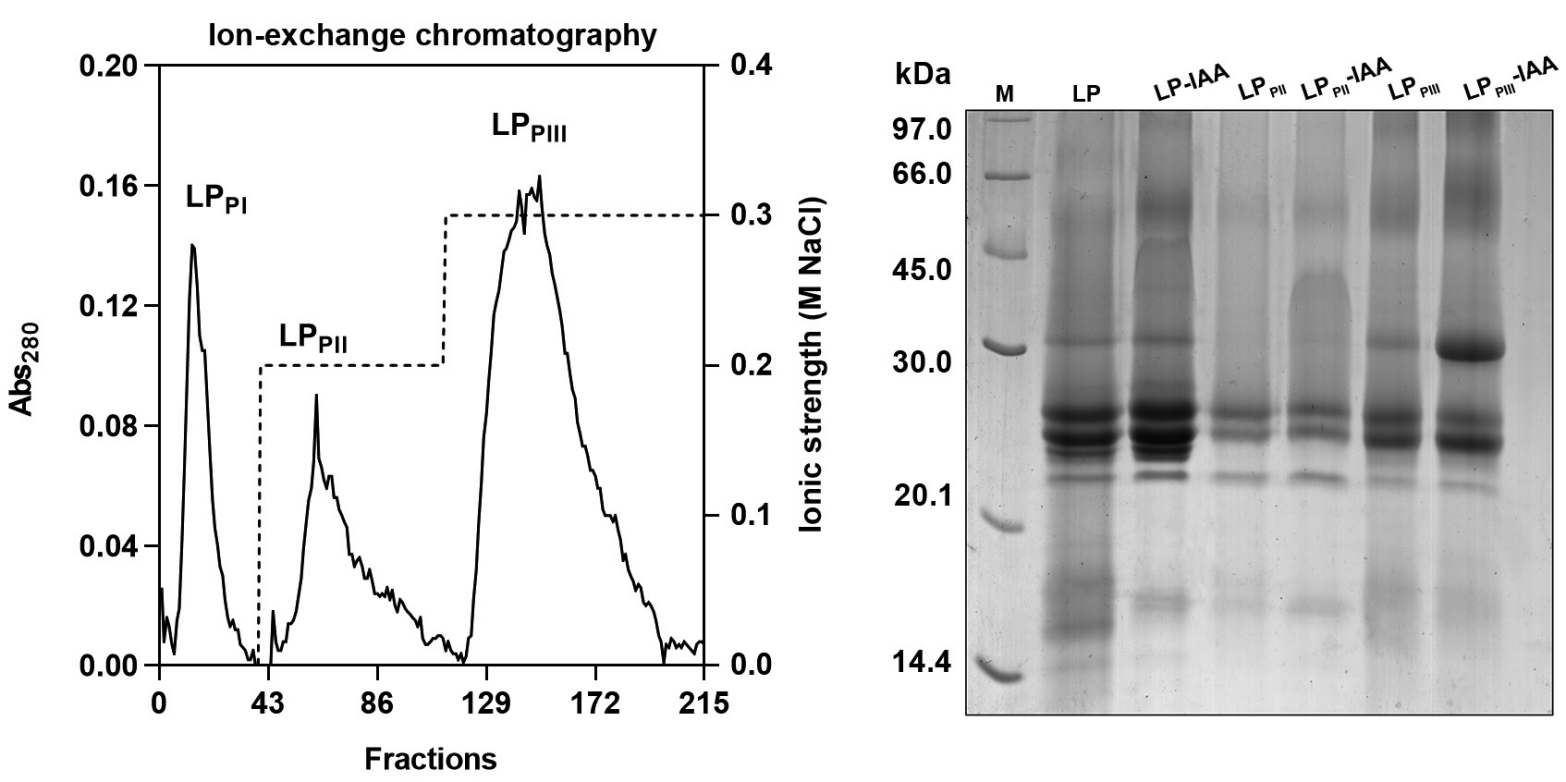
chromatography and sodium dodecyl sulphate-polyacrylamide gel electrophoresis (SDS-PAGE) of *Calotropis procera* LP and its subfractions. The panel on the left contains an Ion-exchange chromatography in CM-Sepharose Fast Flow column performed at pH 5.0 of *C. procera* LP. Subfractions LP_PII_ and LP_PIII_ were eluted by increasing ionic strength by adding 0.2 M and 0.3 M of NaCl to elution buffer, respectively. The right panel shows the protein profiles of LP, LP_PII_ and LP_PIII_ and they after iodoacetamide (IAA) treatment. M: molecular weight markers: 97.0 kDa: phosphorylase β; 66.0 kDa: albumin; 45.0 kDa: ovalbumin; 30.0 kDa: carbonic anhydrase; 20.1 kDa: trypsin inhibitor and 14.4 kDa: α-lactalbumin.

*Latex collection, processing, and fractionation* - The latex extraction, processing and fractionation were successful as shown by the chromatography profile and SDS-PAGE (Fig. 1). LP has at least seven major distinguishable proteins, while LP_PII_ has three major distinguishable proteins and LP_PIII_ has four. The subfraction LP_PIII_ exhibits a well-distinguished major protein, not detectable in LP_PII_, with relative molecular mass near to 30 kDa. The treatment with IAA did not change the protein profile of any of the samples (Fig. 1). This are confirmatory results already available in the preceding literature^12^ that ensure the identification of each preparation.

*Survival evaluation of LP*_*PII*_ *and LP*_*PIII*_ *administration* - In all infected animals that did not receive treatment or that were treated with LP_PII_ or LP_PII_-IAA, it was observed behavioral changes and distress, such as hunched posture, placid behavior, decreased mobility, pointed ears and orbital tightening. However, survived animals treated with LP_PIII_ or LP_PIII_-IAA did not show behavioral alterations.

**Fig. 2:**
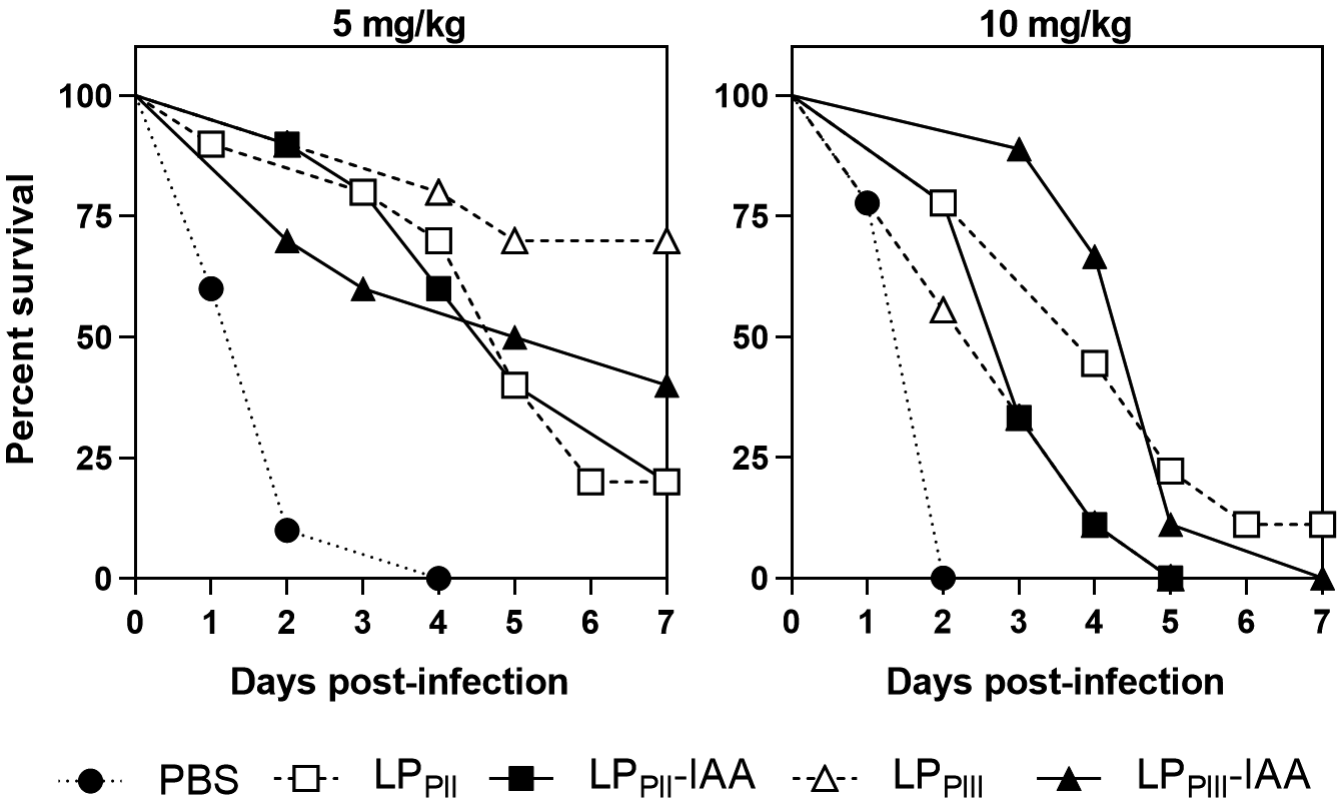
time-course of survival of animals treated with LP subfractions against lethal infection of *Salmonella enterica* serovar Typhimurium. Animals were treated intraperitoneally with LP_PII_, LP_PII_-IAA, LP_PIII_ or LP_PIII_-IAA (5 and 10 mg/ kg) 24 h before bacterial inoculation 0.2 mL, 10^6^ colony-forming unit (CFU)/mL. Animals were observed for seven days. (p < 0.05; n = 10; Mantel-Cox log rank test).

All animals from infected PBS group died within 72 h. The i.p. administration of LP_PII_ and LP_PIII_ increased the resistance of the animals to the lethal bacterial infection, in different degrees (Fig. 2). The animal group treated with LP_PIII_ (5 mg/kg) showed 70% survival rate after seven days, followed by LP_PIII_-IAA (5 mg/kg), with 40% survival. Treatment with either LP_PII_ or LP_PII_-IAA was less efficient, with only 20% survival. The increase in concentration to 10 mg/kg reduced survival in all groups, after seven days. In sight of these results, further experimentations were carried out using LP_PIII_ (5 mg/kg).

**Fig. 3:**
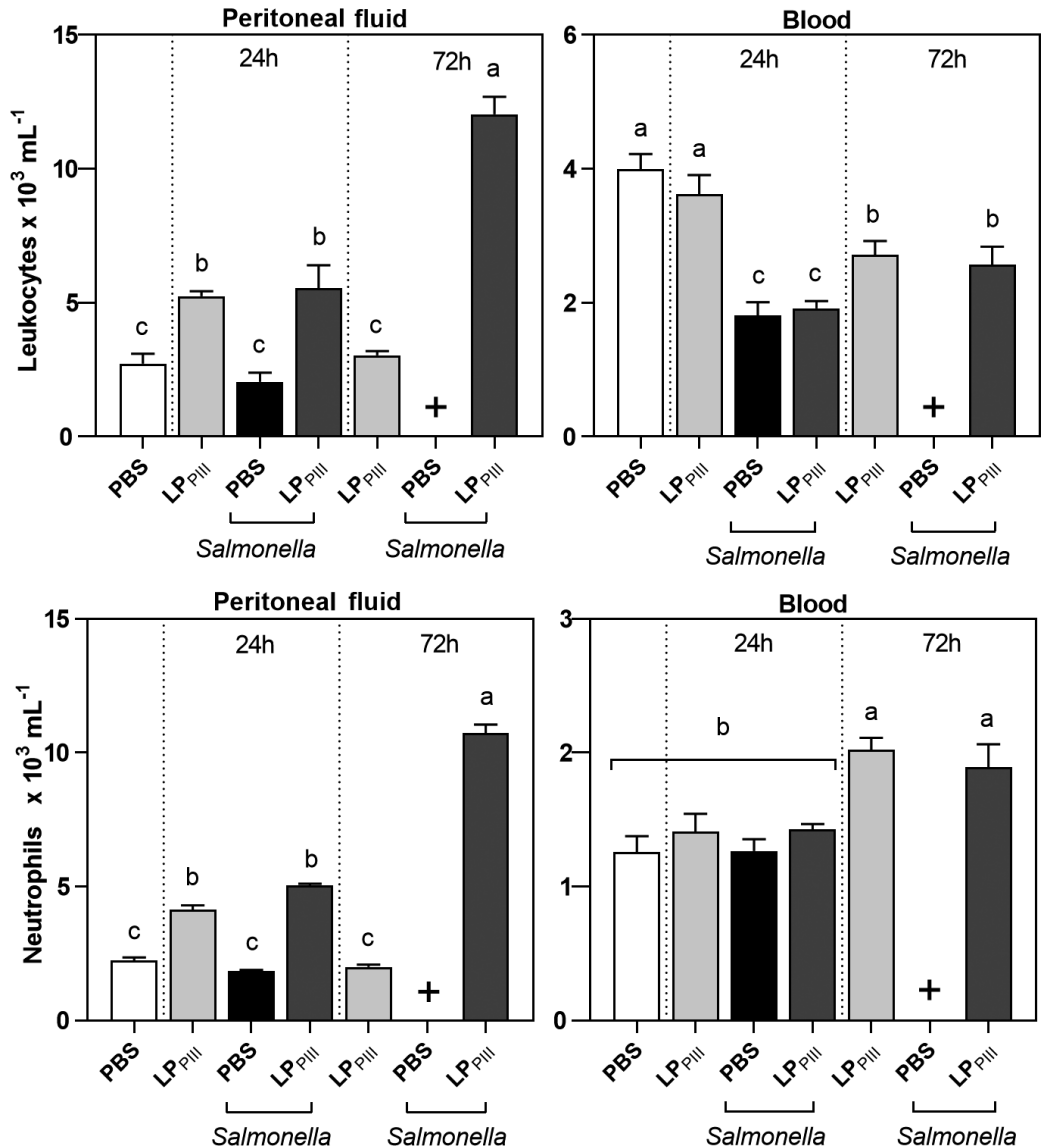
leukocyte and neutrophil count in the peritoneal cavity and blood. Swiss mice were treated (i.p.) with *Calotropis procera* LP_PIII_ (5 mg/ kg) 24 h before *Salmonella* Typhimurium inoculation 0.2 mL, 10^6^ colony-forming unit (CFU)/mL. Leukocyte and neutrophil enumeration were evaluated 24 and 72 h after infection. The (+) sign indicated that all animals from that group died. Results are expressed as mean ± standard error of mean (SEM) cells. Different letters indicate statistically significant differences (p < 0.05; n = 8; one-way analysis of variance (ANOVA) - Bonferroni test).

*Effect of treatment with LP*_*PIII*_ *over cell immunity in face of bacterial infection* - In the peritoneal cavity (Fig. 3), the administration of LP_PIII_ induced a leukocyte migration in both, healthy and infected mice. However, this effect was transient in healthy and only detected within 24 h while it was 2-fold increased after 72 h in infected animals. In the blood (Fig. 3), the administration of LP_PIII_ in healthy mice caused a slight leukopenia only detected within 72 h. During infection, the animals that received LP_PIII_, had a mild leukopenia within 24 h, similar to the infected PBS group (Fig. 3).

When comparing the number of neutrophils (Fig. 3) to the total number of leukocytes in the peritoneal cavity, it is noted that more than 85% of those leukocytes were neutrophils in every group, meaning that i.p. administration of LP_PIII_ caused neutrophil infiltration to the peritoneal cavity after 24 and 72 h of infection. When analysing the number of neutrophils in the blood, no change was observed in any group within 24 h of infection. However, there was a decrease in total leukocyte counts in infected groups within 24 h, meaning that other white blood cells, such as lymphocytes, decreased in number, this relative lymphopenia is reported in cases of typhoid fever.^16^

The i.p. administration of LP_PIII_ did not alter leukocyte in the blood but increased in the peritoneal cavity of healthy animals. However, this effect increased within 24 and 72 h in infected animals, suggesting that LP_PIII_ also played a role in activating the later inflammatory response.^17^

**Fig. 4:**
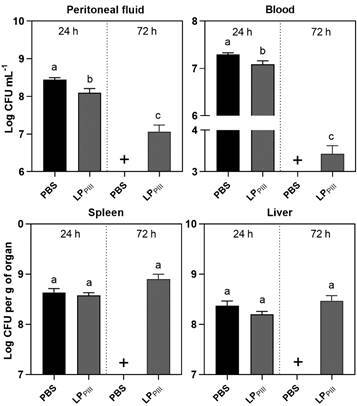
bacterial enumeration. Animals were treated with LP_PIII_ from *Calotropis procera* (5 mg/kg) 24 h before lethal infection with *Salmonella* Typhimurium suspension 0.2 mL, 10^6^ colony-forming unit (CFU)/mL. 24- and 72-hours post-infection, blood, peritoneal fluid, spleen, and liver were aseptically collected; fluids were diluted, and organs were homogenised in phosphate-buffered saline (PBS) and plated in Petri dishes with sterile MacConkey agar medium. After 24 h of incubation at 37ºC, the number of CFU was identified. Values are expressed as mean ± standard error of mean (SEM) of CFU per milliliter or per gram of organ. The (+) sign indicates that all animals from that group died. Different letters indicate statistically significant differences (p < 0.05; n = 8, one-way analysis of variance (ANOVA) - Bonferroni test).

Corroborating with the increase in neutrophil numbers in the peritoneal cavity, the treatment with LP_PIII_ caused significant reduction in bacterial load in the peritoneal fluid within 24 h, furthermore, after 72 h there was a consistent decrease in bacteria enumeration (3 - 4 Log CFU/mL) in the peritoneal cavity, compared to the infected PBS group of animals (Fig. 4). A significant reduction in bacterial load was also observed in the blood after 72 h. The early recruitment of neutrophils caused by LP_PIII_ primed the cellular immunity in the site of infection, reducing the bacterial load. This effect probably contributed to protecting the animals against septic shock, and this is well represented in the survival rate observed. As seen in past studies, the numbers of *S.* Typhimurium CFU increases in the organs that are their target of the infection, such as spleen and liver.^18^ The results observed in Fig. 4 suggest that despite the initial reduction of bacteria load in the peritoneal cavity of animals treated with LP_PIII_, the load of bacteria in liver and spleen was similar among all survival animals (24 or 72 h). Therefore, the chemiotactic effect displayed by LP_PIII_ over neutrophils was not pivotal to abrogate infection.

**Fig. 5:**
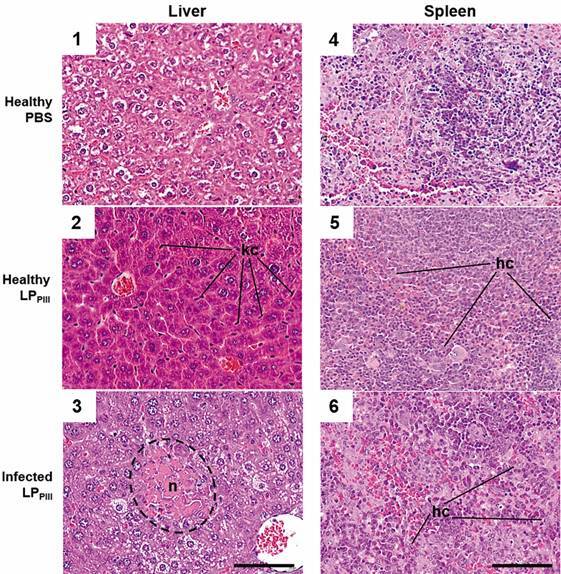
images of microscopic slides of liver and spleen of healthy animals and infected animal treated or not with LP_PIII_. The most representative images of these groups were chosen. Animals were euthanised and the organs were aseptically removed. After fixation and inclusion and paraffin, 5 µm cuts were done in a microtome. The cuts were fixated in microscope slides and stained with hematoxylin and eosin for the analysis of inflammatory infiltration and organ architectural damage. kc: Kupffer cells; n: necrosis; hc: histiocytes.

*Histopathology* - LP_PIII_ caused moderate inflammatory swelling in liver and spleen. Mononuclear phagocytic system cells were detected in higher than normal amounts, such as Kupffer cells and histiocytes in the liver and spleen, respectively (Fig. 5). Within 24 h of infection, the animals of PBS group showed signs of intense hydropic degeneration in the liver and focal apoptosis in the spleen, both organs had inflammatory infiltrates. After 72 h various sites of apoptosis were detected in liver and spleen. Twenty-four hours after infection, animals of the LP_PIII_ group exhibited highly spread Kupffer hyperplasia and histiocytosis in the liver and spleen, respectively. After 72 h, there was a high number of neutrophilic infiltrations in the liver and moderate apoptosis in both organs.

Kupffer cells has a pivotal role in starting and controlling systemic inflammation. Kupffer cells release IL-10, an anti-inflammatory cytokine that quickly disperse in the circulation through the hepatic vasculature. Besides that, these cells can neutralise bacteria by means of nitric oxide and reactive oxygen species, serving as an important barrier against invasive bacteria such as *S.* Typhimurium^19^ The increase in Kupffer cells in liver, caused by LP_PIII_ treatment probably afforded means to the control of bacterial load in the organs, and helped increasing circulating levels of IL-10.

**Fig. 6:**
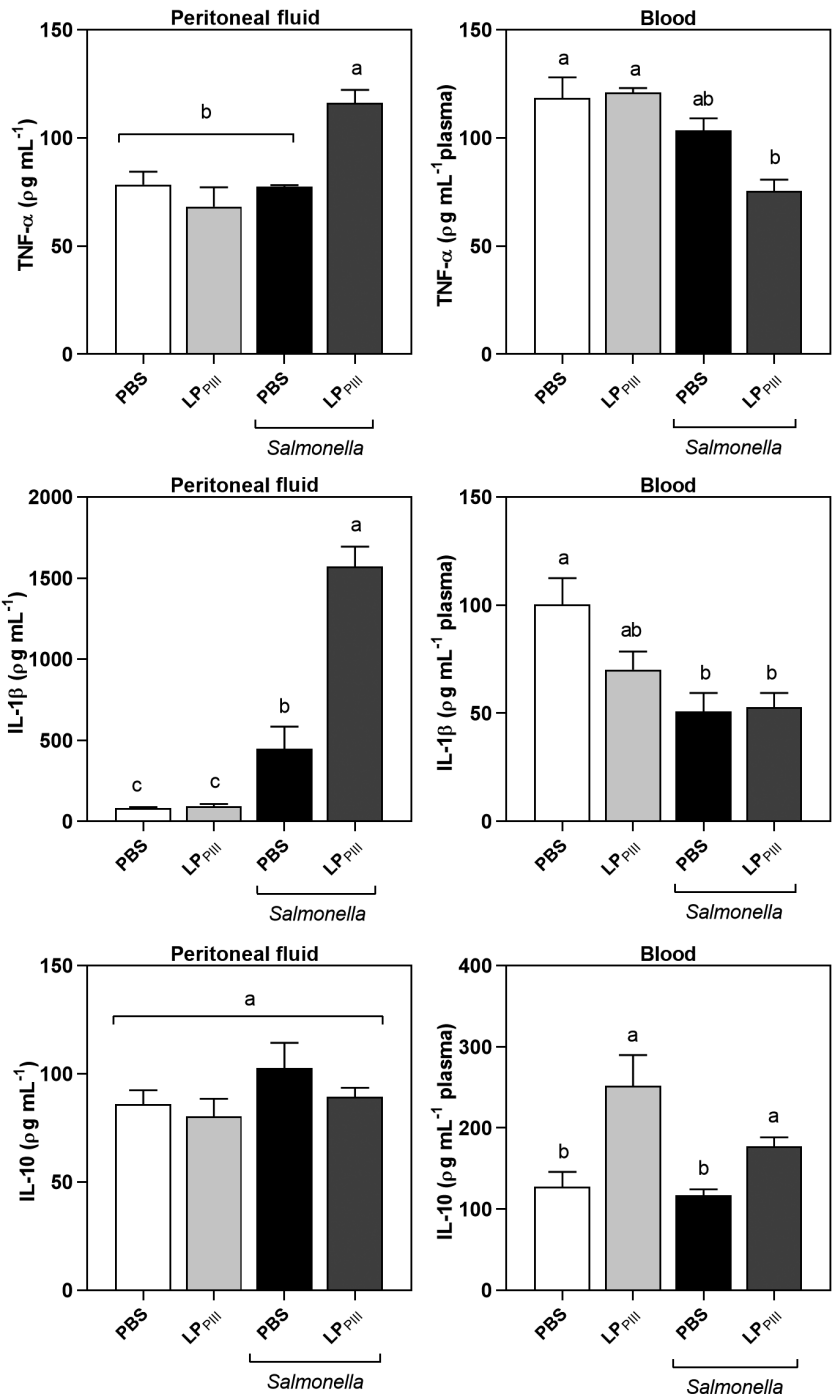
measurement of TNF-α, IL-1β and IL-10 in the peritoneal fluid and blood plasma of mice treated or not with LP_PIII_, infected or not by *Salmonella*. After 24 h of infection, blood and peritoneal fluid were collected and the cytokines were measured using mouse enzyme-linked immunosorbent assay (ELISA) kits. Values are expressed as mean ± standard error of mean (SEM) of ρg of cytokine per mL of fluid or plasma. The (+) sign indicates that all animals from that group died. Different letters indicate statistically significant differences (p < 0.05; n = 8, one-way analysis of variance (ANOVA) - Bonferroni test).

*Cytokines* - The cytokines TNF-α, IL-1β and IL-10 were measured due to their central role in the inflammatory process. The levels of TNF-α did not change neither 24 h after LP_PIII_ administration in healthy animals, nor 24 h after bacterial infection, both in the peritoneal cavity and in the blood. However, TNF-α concentration in the peritoneal fluid was significantly higher in animals that received the protein administration before the infection, although in the blood, this same group exhibited significantly lower levels of TNF-α (Fig. 6). The levels of IL-1β in the peritoneal fluid of healthy animals treated with LP_PIII_ did not change. During infection, the PBS group had a 5-fold increase in concentration, while the LP_PIII_ group exhibited a 16-fold increase. In the blood, IL-1β levels decreased in healthy LP_PIII_ group. During infection, the concentration of IL-1β decreased significantly for PBS and LP_PIII_ groups (Fig. 6). There was no change in IL-10 in the peritoneal cavity of the different groups. However, in the blood, the administration of LP_PIII_ in healthy and infected animals caused a significant increase in the cytokine’s level (Fig. 6). This increase may be related to the increase in Kupffer cells caused by LP_PIII_ administration.

**Fig. 7:**
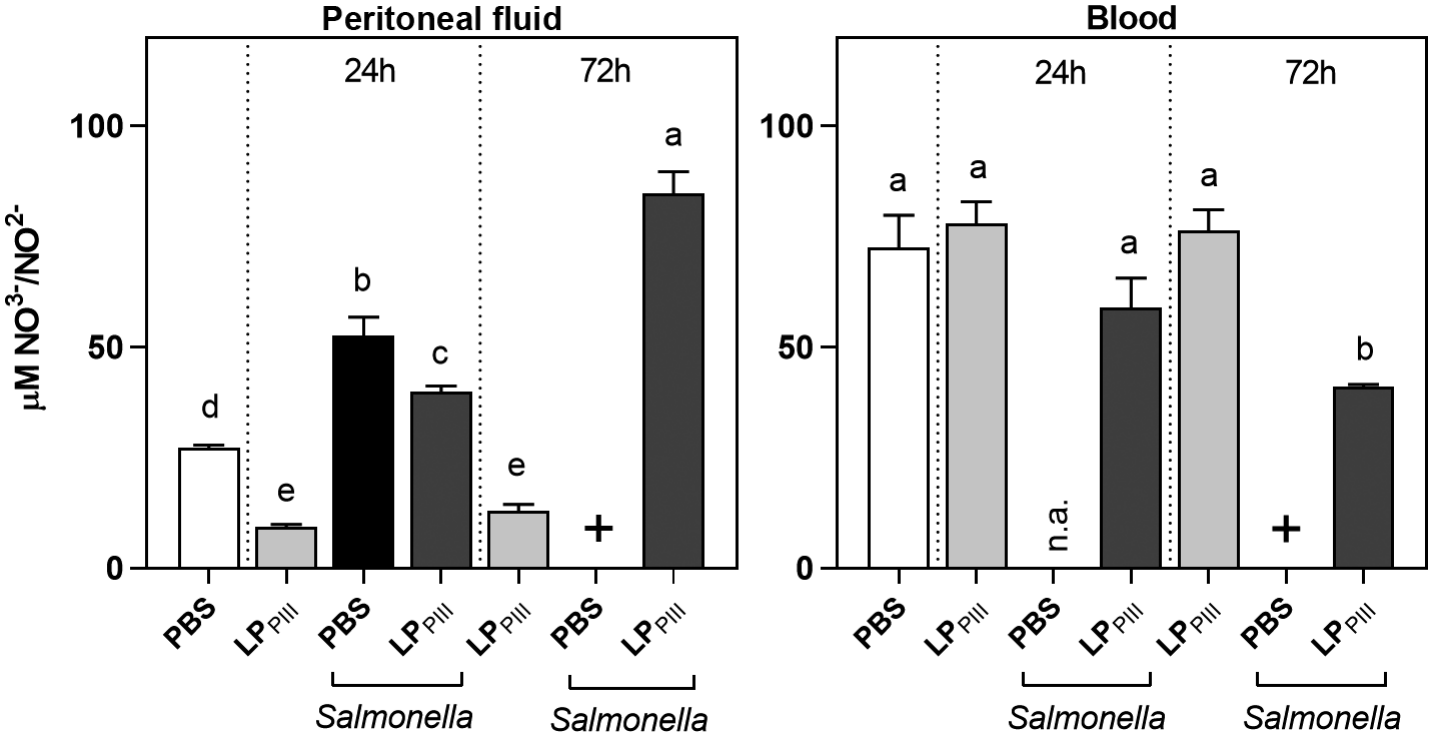
indirect measurement of nitric oxide (NO) by detecting NO^3-^, in the peritoneal fluid and blood plasma of treated and untreated, infected, and uninfected mice. Animals received *Calotropis procera* LP_PIII_ (5 mg/kg) 24 h before infection with *Salmonella* Typhimurium suspension 0.2 mL, 10^6^ colony-forming unit (CFU)/mL. Blood and peritoneal fluids were collected 24 h and 72 h after infection. Values are expressed as mean ± standard error of mean (SEM) of µM of NO^3-^/NO^2-^. Labels (n.a.) indicates that data from that group was not available due to experimental constrains. The (+) sign indicates that all animals from that group died. Different letters indicate statistically significant differences (p < 0.05; n = 8, one-way analysis of variance (ANOVA) - Bonferroni test).

*Nitric oxide* - In the peritoneal cavity, the administration of LP_PIII_ lowered the NO release within 24 h. After 72 h, the concentration remained the same in these animals. During infection, infected-only animals had higher concentrations of this inflammation mediator in the peritoneal cavity, while treated and infected animals exhibited valued lower than the infected-only group but higher than the control. After 72 h, the NO levels in treated and infected animals exhibited significantly higher concentrations (Fig. 7). In the blood, it is observed that the administration of LP_PIII_ did not change the levels of NO in healthy animals that received the protein i.p. Treated and infected animals did not exhibit changes in NO concentration within 24 h, but after 72 h it was lowered (Fig. 7). The treatment with LP_PIII_ lowered the NO levels in healthy mice. This reduction also happened during infection that probably helped controlling the oxidative stress.

**Fig. 8:**
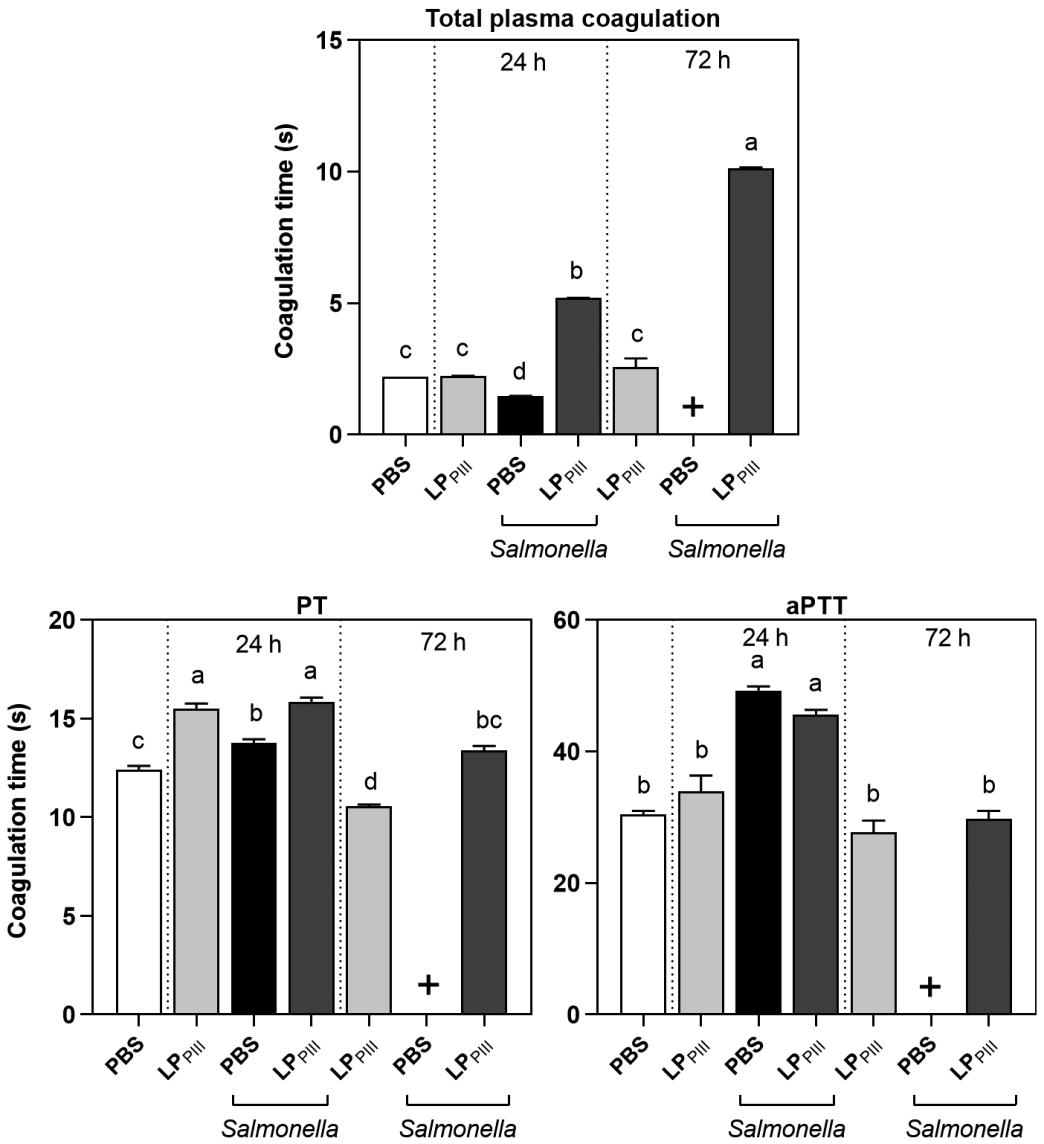
effect of LP_PIII_ from *Calotropis procera* on total plasma coagulation and involvement in prothrombin (PT) and activated partial thromboplastin time (aPTT) in infected and uninfected Swiss mice. Animals were treated by intraperitoneal route with 5 mg/kg LP_PIII_ 24 h before infection 0.2 mL, 10^6^ colony-forming unit (CFU)/mL. After 24- and 72-hours post-infection, plasma was collected, and coagulation time essays were executed following manufacturer’s instructions. Coagulation time was measured as the time between the addition of CaCl_2_ and the detection of clot formation. Data were expressed as mean ± standard error of mean (SEM) of coagulation time in seconds. The (+) sign indicates that all animals from that group died. Different letters indicate statistically significant differences (p < 0.05; n = 8, one-way analysis of variance (ANOVA) - Bonferroni test).

*Plasma coagulation* - The treatment with LP_PIII_ in healthy mice did not alter blood coagulation time. During infection, the animals of the PBS group had a reduction in total plasma coagulation time, while LP_PIII_ group strongly slowed the coagulation time within 24 h and 72 h (Fig. 8). The treatment only had an effect over plasma coagulation during the infection. To further investigate the effect of the treatment over different coagulation cascades, PT and aPTT assays were executed.

In PT assay, the treatment with LP_PIII_ increased coagulation time in healthy and infected animals within 24 h. In aPTT assay, the treatment did not change the coagulation time of healthy or infected animals. This suggests that the effect of LP_PIII_ over the plasma coagulation happened in the extrinsic pathway that is dependent on tissue factor signaling. During chronic bacterial infections, the disseminated intravascular coagulation starts via tissue factor signaling.^20^

Altered levels of inflammatory cytokines, neutrophils and macrophages indicate whether an acute inflammatory process is established. This scenario activates the tissue factor signaling pathway. However, as shown in these results, the treatment with LP_PIII_ created a local and brief inflammatory response and a later systemic anti-inflammation signaling, through IL-10 release in the blood. Both events seemed to contribute to face the spread of the bacteria infection through the blood, avoiding the activation of the extrinsic coagulation pathway.

## DISCUSSION

This study contributes to the knowledge of the role of *C. procera* LP in modulating the inflammatory process during infections mediated by bacteria. The results presented here demonstrated that despite of the total latex proteins fraction from *C. procera* afford protection to infected animals, this action was not restricted to the subfraction LP_PI_, as described previously. LP_PIII_ on the other hand, was demonstrated here that it was also effective in helping animals to overcome the experimental acute bacterial infection.

Furthermore, here it was demonstrated that the protection afforded by LP_PIII_ was statistically similar to LP_PIII_ treated with IAA, a classical reducing agent of disulfide bonds. IAA irreversible inhibits proteolysis of cysteine peptidases. However, it also reduces cysteine residues not involved in proteolysis and potentially disrupt protein structures. This observation suggested that the effectiveness of LP_PIII_ was not related to proteolysis. Further experimental approaches are needed to a better comprehension of this aspect.

The single administration (i.p.) of LP_PIII_ in healthy animals induced neutrophil migration to the administration locus. Thus, at the beginning of the infectious process, LP_PIII_ may have facilitated phagocytosis, by recruiting inflammatory cells. The chemotactic effect of LP_PIII_ over neutrophils resulted in bactericidal activity mediated by NO release. Also, it is expected that activated neutrophils forwarded the immune response through pro-inflammatory cascade. However, bacterium was not cleared in spleen and liver. In these target organs, the bacteria caused moderate histological damage. The infiltration of phagocytic cells, detected in these organs, was due LP_PIII_ treatment. Therefore, this event possible triggered expression and release of IL-10 in the blood. Elevated level of IL-10 in the blood may have a pivotal role in controlling inadequate inflammation, typically observed in the course of uncontrolled bacterial infections.

TNF-α signals many cell functions critical to the inflammatory response such as proliferation, differentiation, survival, and apoptosis. It is mainly produced by macrophages, which are highly sensitive to TNF-α. This cytokine plays a regulator role in commanding other inflammatory cytokines production.^21^ The *C. procera* LP and LP_PI_ administration in the peritoneal cavity was shown to induce TNF-α production within 4 h post-infection, due to the induction of NO, while non treated and infected animals had a delayed production of this cytokine.^6^ In our experiment, the administration of LP_PIII_ did not cause release of TNF-α, detected at 24 h, in the peritoneal cavity.

Although TNF-α plays a pivotal role in initiating the inflammatory response, other cytokines oversee the persistence of the inflammation. For example, TNF acts as transcriptional regulator of inflammasome components. An inflammasome is a protein complex that is responsible of producing the active mature form of inflammatory cytokines, such as IL-1β.^22^ Here, it was demonstrated that animals infected with *Salmonella* exhibited significantly high levels of IL-1β in the peritoneal cavity, coming from the inflammatory response against the infection.^23^ This cytokine will activate inflammatory tissue and recruit macrophages and neutrophils, similar to observed with LP_PI_.^3^

*Salmonella* causes the release of chemokines and cytokines that provoke a local inflammatory response, attracting neutrophils and macrophages to use as a new host to disseminate itself through the body. Whereas *Salmonella* survives inside macrophages, neutrophils are able to kill the bacteria, due to the high concentration of ROS in the phagosome of these cells.^24^ In fact, the neutrophil numbers in animals treated with LP_PIII_ increased significantly, coordinated with the release of IL-1β, that acted recruiting these cells, resulting in the reduction in bacteria numbers at the site of infection.

Among the cytokines produced during severe bacteremia, IL-10 acts as an immunoregulatory molecule. It is produced by various leukocytes including neutrophils and T cells, and it down-regulates signaling receptors that reverts the inflammation progress, including halting immune cell proliferation, antigen presentation and inhibition of oxidative burst. During bacterial infection, the time at which IL-10 is produced and released may have harmful results and contribute to organ damage, reduced bacterial clearance and overall lower survival rates, because the inhibition of the inflammatory response can allow pathogens to escape immune control, causing rapid dissemination of the pathogen to vital organs resulting in fatal infections.^25^ In this experiment, LP_PIII_ caused an increase in IL-10 in the blood regardless healthy or infected animals. This early release of circulating IL-10 in the blood was essential in avoiding the exacerbation of the inflammation caused by *Salmonella*, allowing more time for leukocyte migration and phagocytosis of bacteria. However, high levels of this cytokine impairs pathogen clearance while ameliorating organ damage.^25^ This effect was seen in this study, in animals that received LP_PIII_, bacteria levels did not decrease in organs, while the level of tissue disarray was lower compared to animals of infected PBS group.

The inflammatory response, mediated by immune cells, against severe and uncontrolled infections, results in cell death and organ damage, and ultimately the disseminated intravascular coagulation: the septic shock. NO is a signaling molecule that have defensive roles against infectious diseases but also has harmful effects over the organism.^26^ NO was found to be essential in enhancing survival of mice infected by *Salmonella* Typhimurium.^27^ In this study, NO levels were reduced in healthy animals treated with LP_PIII_. This reduction was also observed in treated infected animals. Therefore, LP_PIII_ also contributed to modulate NO release. The control of the oxidative stress during the infection prevented further damage and exacerbation of the inflammation.

The same inflammatory regulators which result in a systemic response, also activates the coagulation system. Previous studies have demonstrated that *C. procera* LP reduces the clotting time^10^ and is able to change the fluid nature of the blood from pseudoplastic to Bingham fluid which means that it reduces the blood fluidity increasing its viscosity. However, these effects seem to be transient and do not lead animals to death.^28^ Here, it was used a subfraction of that sample and it was not observed any alteration in the total blood coagulation time in healthy animals. The infection caused the reduction in coagulation time. Treated-infected animals exhibited higher coagulation time. Moreover, the administration of LP_PIII_ in healthy animals increased extrinsic coagulation time. This reveals that the influence of *C. procera* LP over the coagulation system is dependent on the subfraction analysed and that LP_PIII_ has an action only over the tissue factor (TF) signaling coagulation pathway. A prolonged PT is related to levels of factor VII, its synthesis can be reduced due to liver damage or increased consumption, due to coagulopathies.^29^

In addition, LP_PIII_ was previously described as containing peptidases with both thrombin- and plasmin-like activities *in vitro*.^10^ It was demonstrated here that this subfraction has an anti-coagulant activity *in vivo*, during the infectious process, by increasing coagulation time in infected animals. When analysing extrinsic and intrinsic coagulation pathways, the protein inoculation likely affected TF signaling, due to the increase in prothrombin coagulation time 24 h after infection. TF is produced by activated macrophages and monocytes, as well as endothelial cells and organ capsules and its expression is upregulated by proinflammatory cytokines.^20^

In conclusion, LP_PIII_ demonstrated to afford protection against lethal infection induced by *Salmonella*. As previously observed for LP_PI_, the survival of animals treated with LP_PII_ or LP_PIII_ was lower than that observed to LP. Therefore, it is proposed that the full protection observed, when animals are treated with LP, is due synergism effect of different proteins found in the different subtractions of LP (i.e. LP_PI_, LP_PII_, LP_PIII_). Experimental evidences suggest that different contributions, belonging to different proteins, underlying specific pathways of action provide LP the efficiency for full protection against different inflammatory stimulus. In the case of LP_PIII_, the recruitment of inflammatory cells to the primary site of infection seems to play an important role to control infection. The observed increment of the anti-inflammatory IL-10 cytokine, in the blood, would play a pivotal effect against bacterial lethality. Lastly, the observation that LP_PIII_ contributes to slow down blood coagulation in septic animals, through modulation of the extrinsic pathway, would explain the absence of clinical signs of disseminated intravascular coagulation, the ultimate physiological event documented in septic death. The pathway leading to IL-10 production through LP_PIII_ treatment remains fully unknown.
